# Towards Automatic UAS-Based Snow-Field Monitoring for Microclimate Research

**DOI:** 10.3390/s19081945

**Published:** 2019-04-25

**Authors:** Petr Gabrlik, Premysl Janata, Ludek Zalud, Josef Harcarik

**Affiliations:** 1 Central European Institute of Technology, Brno University of Technology, Purkynova 123, 612 00 Brno, Czech Republic; ludek.zalud@ceitec.vutbr.cz; 2 The Krkonose Mountains National Park Administration, Dobrovskeho 3, 543 01 Vrchlabi, Czech Republic; pjanata@krnap.cz (P.J.); jharcarik@krnap.cz (J.H.)

**Keywords:** snow mapping, UAS, photogrammetry, remote sensing, direct georeferencing, snow field, snow-covered area, snow depth

## Abstract

This article presents unmanned aerial system (UAS)-based photogrammetry as an efficient method for the estimation of snow-field parameters, including snow depth, volume, and snow-covered area. Unlike similar studies employing UASs, this method benefits from the rapid development of compact, high-accuracy global navigation satellite system (GNSS) receivers. Our custom-built, multi-sensor system for UAS photogrammetry facilitates attaining centimeter- to decimeter-level object accuracy without deploying ground control points; this technique is generally known as direct georeferencing. The method was demonstrated at Mapa Republiky, a snow field located in the Krkonose, a mountain range in the Czech Republic. The location has attracted the interest of scientists due to its specific characteristics; multiple approaches to snow-field parameter estimation have thus been employed in that area to date. According to the results achieved within this study, the proposed method can be considered the optimum solution since it not only attains superior density and spatial object accuracy (approximately one decimeter) but also significantly reduces the data collection time and, above all, eliminates field work to markedly reduce the health risks associated with avalanches.

## 1. Introduction

Environmental mapping embodies a relevant target field for unmanned aerial system (UAS)-based photogrammetry. The low cost, safety, and flexibility of operation allow us to employ aerial mapping in domains where manned aircraft cannot be used profitably. One of the possible applications consists of snow-cover mapping, which is beneficial within, for example, avalanche and flood forecasting, local and regional climate research, and hydropower energy situation analysis. Dependable information about snow conditions is especially important for northern countries, where the snow cover is present for a significant part of the year.

There are various methods to estimate certain snow-cover parameters, and each of these techniques is beneficial at a different scale and in diverse applications. The basic parameter rests in determining the presence of snow, namely the snow-covered area (SCA). Such information is of interest mainly for the investigation of trends in large areas (or, more concretely, at the level of regions and countries) and finds use in climate and hydrological research. In general, terms, two determining approaches are typically employed: optical and microwave-based survey. Snow coverage is recognizable from aerial imagery (collected from manned aircraft or satellites) only with a clear, cloudless view [1,2]. Conversely, radar-based observation is independent of the weather conditions, although the resolution and accuracy are typically lower [2,3,4]. In any case, the information value of the SCA is limited because it does not describe the amount of snow or the relevant content of water.

The snow depth (SD) indicator provides us with a better insight into the amount of the accumulated snow. The SD in a certain area can be estimated via either interpolating data from a network of observation stations [5] or as a result of a fusion of point measurements and satellite-based observations [6]. In certain conditions, SD is also estimable from radar-based measurements or by using the light detection and ranging (LiDAR) technology; terrestrial and airborne laser scanning (TLS, ALS) have been recently used for this purpose [7,8,9]. The most meaningful indicator in this context is the snow water equivalent (SWE), describing the amount of water contained in the snow cover. This type of information is crucial for hydrologists, especially as regards flood prediction during snow melting periods. As with SD estimation, the SWE is frequently obtained from in situ measurements. The spatial resolution is increased either via fusing space-born radiometric measurements [10] and ground observations [11] or by using snow models [4].

Manned aircraft and satellite-based estimation of the snow-cover parameters are not suitable for small areas and applications that require high accuracy, the reasons being the low resolution, low estimation accuracy, and substantial cost. Considering other relevant approaches, the resolution of the point measurements is not sufficient due to the sparse network of observation stations. In such cases, unmanned aircraft can be employed effectively. Micro and light UASs (below 5 kg and 50 kg [12], respectively), the categories addressed within this study, are profitably employed in many fields of aerial mapping, such as agriculture [13], forestry [14], geodesy [15], archaeology [16], or environmental mapping [17]. However, due to their limited endurance, sensitivity to weather conditions, and relevant legal restrictions, the vehicles are not suitable for global area mapping, including flights at the regional level. Conversely, the concept ensures fast, safe, low-cost, and flexible operation, and it enables us to reach superior accuracy and resolution.

Most of the recent UAS-based snow mapping projects use aerial photogrammetry for SD estimation. Studies [7,8,18], for example, compare photogrammetry and indirect georeferencing-based SD estimation with terrestrial laser scanning in mountainous regions. The results indicate that both methods are suitable for the given purpose and achieve comparable accuracies. Indirect georeferencing (IG), a technique relying on ground targets, can be successfully replaced with visual landmark-based co-registration; however, as pointed out within sources [19,20,21], common visible points must exist in both snow-covered and snow-free scenes. According to [7,18], photogrammetric accuracy in snow mapping can be enhanced using near-infrared (NIR) cameras instead of visible-spectrum ones since the former perform well even in weak lighting conditions. In terms of the UAS type, all the referenced projects (except for [7]) use multi-copters to carry out the discussed photogrammetric task, mainly because of their greater wind resistance and higher payload capacity. The articles further indicate that UAS-based snow mapping is appropriate for areas up to tens of thousands of square meters. The idea of the photogrammetry-based depth estimation lies in that the actual surface model of the snow cover is compared with a reference snow-free model. Relevant reference data can be obtained in multiple ways. The common approach is to create the snow-free surface model via the same technique, namely UAS photogrammetry, in a period when there is no snow cover [7,8,18,19,20,21]. Another option rests in using national terrain models (typically produced through LiDAR measurements), which are generally available in many countries.

The SD map is obtained from the height difference between the snow-covered and the snow-free surface models. To acquire such a difference, both models must be accurately georeferenced. All the above-mentioned research projects, except for [21], use IG or visual landmark-based co-registration. IG typically achieves an object accuracy slightly better than that of direct georeferencing (DG) (centimeter to decimeter level); however, a serious health risk may arise during the ground survey due to avalanches, especially in mountainous regions. Despite benefits of the DG, the approach is not widely employed presently. Difficulties addressed in the study [21] and other articles using DG in UAS photogrammetry [22,23,24] relate to the complexity of the hardware equipment onboard UAS. This fact often causes reliability issues. Furthermore, such systems require calibration and precise time synchronization, higher payload capacity, and finally, the overall cost is higher.

As in the case of manned aircraft and satellite-based snow-cover mapping, passive microwave (radar) devices can be carried by UASs as well. Such an approach, discussed within [25], is nevertheless rather uncommon.

This article embodies a case study on UAS-based SD mapping performed with a multi-sensor system specially designed for DG in aerial photogrammetry. The study area, namely the Mapa Republiky snow field, has attracted the interest of scientists because of its particular characteristics; thus, various techniques for estimating SD and SCA have been tested there to this day. Our approach has the potential to reduce health risks and man-performed field work without significant accuracy limitations. The benefits and drawbacks of the presented method are discussed and compared with the previously employed techniques.

## 2. Materials and Methods

### 2.1. Study Area and the Monitoring History

**Figure 1:**
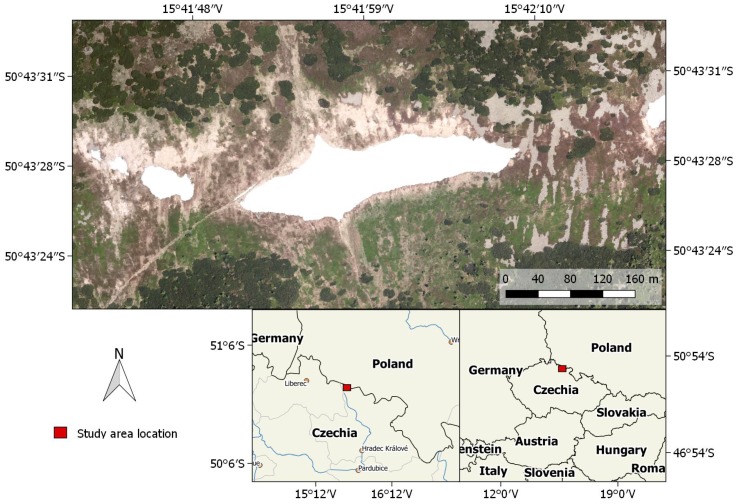
The study site location (the orthophoto obtained during the melting season 2012).

**Figure 2:**
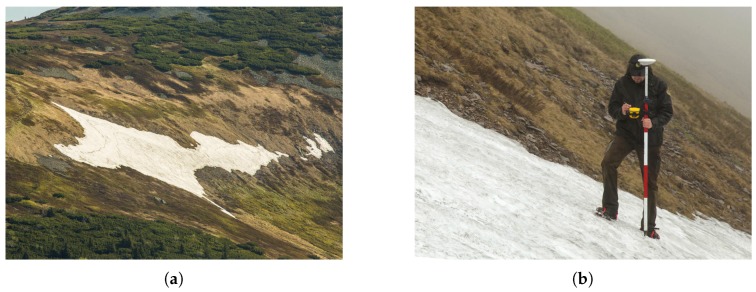
The isolated Mapa Republiky snow field during the spring season (**a**), and ground survey-based point determination of the snow field shape (**b**).

Our method for estimating snow-field parameters was examined at the location called Mapa Republiky (Map of the Republic), a snow field with a shape similar to the outlines of former Czechoslovakia; the area is situated in the Krkonose (Giant Mountains, Figure 1 and Figure 2a). This snow field, at an altitude of approximately 1430 m above mean sea level (AMSL), is rather specific as the snow often persists until the summer season, several months longer compared to other locations in the Krkonose or the Czech Republic in general. This is somewhat unusual, considering the fact that the area is located on a south-facing slope exposed to sunlight during most of the day; the reason rests in the amount of snow contained within the field, where the SD significantly exceeds the average value in the given location (the maximum SD of 15.7 m was established in 2000, Figure 3). The accumulation is caused by northern and northwestern winds depositing snow in the lee. The phenomenon is further intensified by the terrain depression formed by the snow itself [26,27].

**Figure 3:**
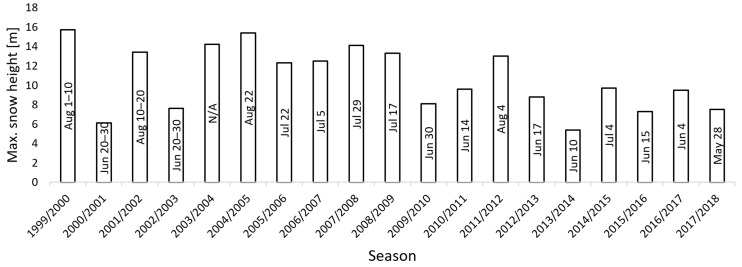
The data on the maximum snow height and melting days since 1999 (the year when the global positioning system (GPS)-based monitoring was performed for the first time).

Depending on the weather conditions during the melting season, together with the precipitation as well as the wind speed and direction in the winter, the snow persists until June to August or, occasionally, does not melt at all (see Figure 3 to read the annual statistics). For this reason, the place is of substantial interest to scientists; the examined problems include, for example, the impact of the SD on the vegetation pattern [26], the relationship between the geo- and biodiversity in the given area [28], and the water balance of the drainage basin. Such analysis, however, requires relevant input information about the amount of snow.

The first attempts to estimate the SD were conducted more than 100 years ago; throughout the 20th century, the research was nevertheless hampered by a lack of appropriate technical equipment [29,30]. Wire probes (collapsible avalanche probes) are not applicable if the snowpack is deeper than 3–4 m, and fixed steel poles may fail due to the shear stress of the snow mass [26]. Systematic investigation was, in fact, made possible only after the introduction of the accurate global positioning system (GPS) technology. Since 1999, the Krkonose Mountains National Park Administration have been monitoring the location via kinematic carrier phase-enabled GPS receivers. The surface of the snow cover is reconstructed by interpolation of GPS position data collected during walking or slow horizontal skiing. These data collection techniques were further supported by the "stop and go" method (walking with stops to facilitate static collection of point data, Figure 2b), which ensured accuracy verification; during periods characterized by a serious avalanche risk, a viable alternative consisted of using a rope-driven sledge with a GPS receiver. Employing the same equipment, data for the construction of a snow-free surface model were collected after the melting season. A SD map was then computed as the difference between these two models. The described approach enabled us to estimate the depth of thick snow layers for the first time [26,31], although the spatial resolution was rather low with respect to the amount of time needed for the data collection.

Another turning point came with the use of UASs. Since 2016, a camera-equipped UAS has been used to acquire relevant aerial image data to be further employed for the photogrammetry-based surface reconstruction. This method significantly reduced the data collection time and, moreover, increased the spatial resolution by two orders of magnitude. Except for the SD, computed in the same manner as within the aforementioned method, this approach enabled us to estimate the SCA employing the actual orthophoto. A major drawback of this method is the necessity of ground targets used for the georeferencing. This task still comprises certain risk due to the avalanche hazard.

### 2.2. Overall Concept

**Figure 4:**
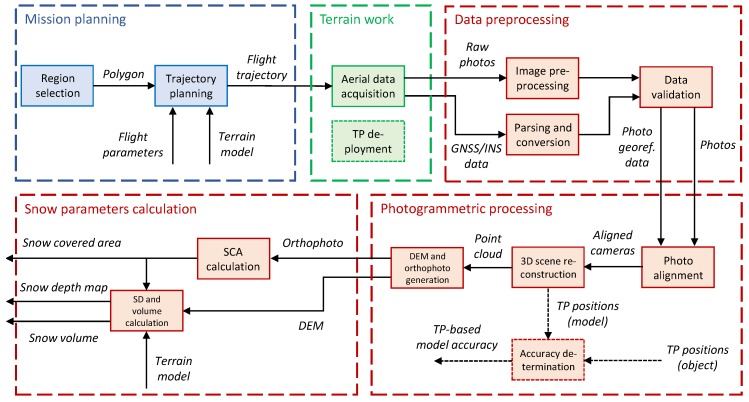
The workflow illustrating the entire snow-field monitoring procedure. TP—test point; GNSS— global navigation satellite system; INS—inertial navigation system; DEM—digital elevation model; SCA—snow-covered area; SD—snow depth.

This paper discusses a vision-based snow-field monitoring method that uses ground control point (GCP)-free UAS photogrammetry (Figure 4). The approach eliminates a substantial portion of the common field work and is thus usable primarily in inaccessible or dangerous areas. Thanks to its capabilities, the technique offers major potential for automated data processing and, importantly, reduces the data acquisition and processing time. Compared to the other relevant methods, however, complex hardware equipment and more specialized operator skills are needed.

Our technique employs aerial photography-based products for SCA calculations; SD and volume calculations nevertheless require another component, namely a snow-free terrain model. Since all aerial data are acquired using a UAS operated in an automatic mode, special attention must be paid to mission planning. This is essential especially in regions with rugged terrain. The field work comprises the aerial data acquisition, or the UAS flight itself, and—if required—test point (TP) deployment. Our UAS is equipped with sensors for DG of the imagery, and thus no ground targets (GCPs) are necessary for the processing. In the proposed experiment, however, we employ several ground targets to enable the previously used IG technique, which will be later compared with our method. By extension, the targets can be used for the DG quality assessment.

The processing part of the workflow is further divisible into three portions. First, the aerial photographs and the data from the global navigation satellite and inertial navigation systems (GNSS and INS, respectively) must be preprocessed and converted into an appropriate format. An important procedure rests in validation, which determines whether the acquired data are consistent and suitable for further processing. The second portion subsumes the photogrammetric processing, comprising tasks well known from UAS photogrammetry, such as the structure-from-motion (SfM)-based 3D scene reconstruction. In our case study, the quality of this process is assessed thanks to the ground targets, or test points (TPs). The orthophoto and digital elevation model (DEM) as photogrammetric products are then used in the final part of the processing to estimate the snow-field parameters. The SCA is computed from the orthophoto, and the SD and volume are estimated primarily from the height differences between the snow-covered and the snow-free terrain models.

Important aspects of the workflow in Figure 4 are addressed in more detail within the following sections. First, the employed UAS and sensors are described in Section 2.3. The ground measurement, a process involving test point deployment (green segment), is then outlined in Section 2.4. Section 2.5 characterizes the mission planning and aerial data acquisition, strongly related topics highlighted in blue and green, respectively. The remaining (red) portions of the Figure display the actual data processing. The first of these segments, data preprocessing, comprises operations necessary for subsequent processing; such steps are not considered in depth, because their relevance to the presented application is limited. The workflow portions comprising photogrammetric processing and snow parameter calculation are examined within Section 2.6 and Section 2.7, respectively.

### 2.3. UAS and Onboard Sensors

To obtain the aerial data, we used a Mikrokopter Ookto XL UAS, a 95 cm span commercial octocopter capable of flying for approximately 10 min with the payload of 2–3 kg. The UAS supports automatic flight based on selected waypoints; the device therefore carries a low-accuracy GPS receiver. The hoverability and high payload capacity are the central reasons why multi-rotor UASs are often employed in photogrammetry missions similar to that outlined herein [24,32,33].

**Figure 5:**
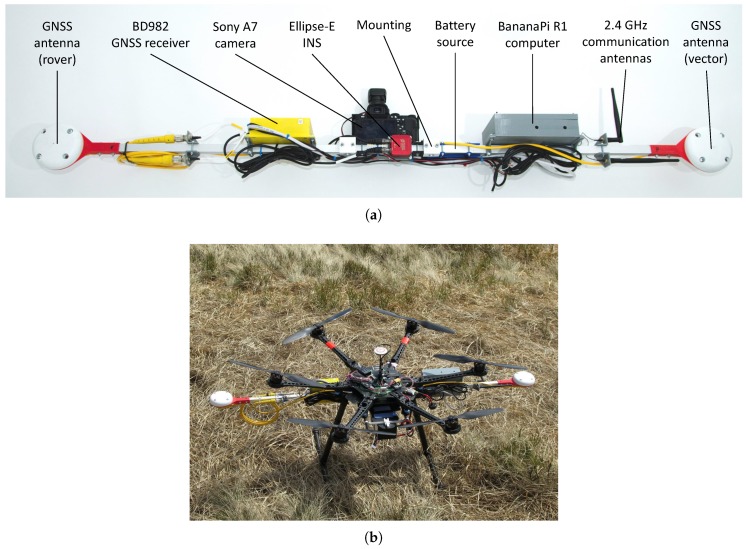
The custom-built multi-sensor system for direct georeferencing (**a**), and a UAS fitted with the system during a previous mapping mission (**b**).

All necessary equipment used for the remote sensing is comprised in the custom-built multi-sensor system mounted on the UAS (Figure 5b). The device was developed previously at CEITEC laboratories and embodies a part of the ATEROS (Autonomous Telepresence Robotic System) robotic system [34,35]. The multi-sensor system consists of a Sony Alpha A7 digital camera, a Trimble BD982 GNSS receiver, an SBG Ellipse-E INS, and a single board computer Banana Pi R1. The GNSS receiver measures the position with centimeter-level accuracy when real-time kinematic (RTK) correction data are transmitted, and as it is equipped with two antennas for vector measurement, the device also measures the orientation around two axes. The position and orientation data are used as an auxiliary input for the INS, which provides data output at a frequency of up to 200 Hz. All the sensors are precisely synchronized; thus, once an image has been captured, the position and orientation data are saved into the onboard solid-state drive (SSD) data storage (more parameters are contained in Table 1).

The aforementioned system is relatively uncommon since it is completely independent of the UAS while integrating all equipment necessary for GCP-free aerial photogrammetry. The relevant testing cycles were performed on various unmanned platforms (such as that visible in Figure 5b) during several missions, with the resulting accuracy assessed in our recent study [36]. Similar setups designed for the DG of aerial imagery but not allowing portability had been published previously [24,32].

The performance of the applied system may satisfy the requirements of the discussed application. With respect to the used SD determination method (Section 2.7), the height accuracy of the photogrammetry-based DEM should be comparable to or better than the snow-free model height accuracy (0.15 m RMSE in our case). Similarly, the horizontal accuracy should reach this level too to prevent height errors caused by inaccurate alignment of the elevation models, an effect apparent especially in steep slopes. The applied multi-sensor system meets such requirements: the object error of the model is typically below a decimeter root mean square (RMS) for both the horizontal and the vertical coordinates when flying at 50 m above ground level (AGL) [36]. In addition to the elevation model, our mapping method uses the actual orthophoto for the SCA calculation (Section 2.7). With respect to the parameters of the used camera and lens (Table 1), the ground sample distances are approximately 14 mm and 29 mm for the AGL altitudes of 50 m and 100 m, respectively. These parameters are far beyond the value necessary for an accurate area estimation in a snow field with a size in the order of hundreds of meters.

### 2.4. Ground Measurements

As already mentioned in the previous section, the proposed concept does not require ground targets for the georeferencing and data processing stages; in fact, the UAS flight is the only field work activity in our concept. Despite this, several ground targets were employed to ensure backward compatibility with the previously used IG technique. Moreover, the targets enable us to determine the accuracy of the directly georeferenced photogrammetric products.

**Figure 6:**
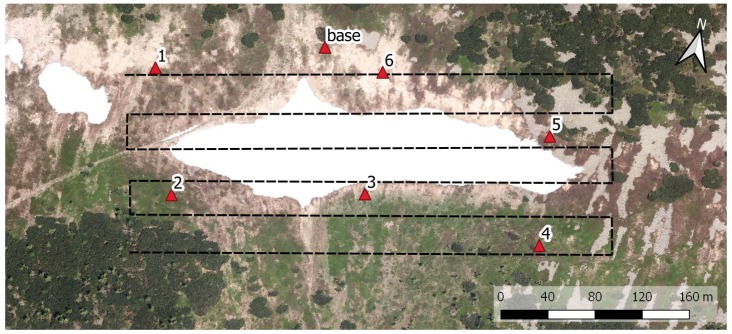
The locations of the ground targets and the base station, together with the flight trajectory for the UAS (the orthophoto obtained during the melting season 2012).

During late April 2018, the time originally selected for our investigation, the Mapa Republiky snow field became completely isolated from the remaining snow cover in the vicinity. This fact allowed the positioning of six ground targets just around the snow field on the peripheral part of the area to be mapped (the distribution is visible in Figure 6). Such a solution was commonly used in the past, enabling us to compare both georeferencing techniques. The horizontal separation of the peripheral GCPs is 140 m on average, or approximately one ground base. The spatial distribution and number of GCPs play a substantial role in the quality of IG in photogrammetry. This aspect has already been addressed within numerous publications [37,38]. Our peripheral location of the targets meets the basic GCP distribution requirement; however, a higher density and extra GCPs located in the central part would help us to increase the georeferencing accuracy.

**Figure 7:**
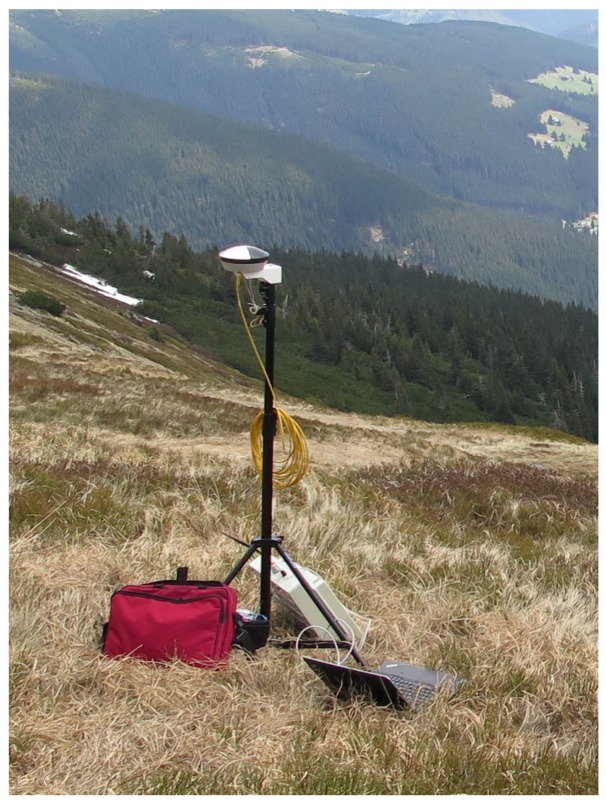
The base station (located close to the snow field) performing real-time GNSS corrections for the sensors aboard the UAS.

We used 20 cm squared, black-and-white-patterned paper targets with clearly defined centers. All these targets were glued onto a solid support and fixed to the ground by using iron nails. The position of every single target was acquired using a survey-grade Topcon HiPer HR RTK GNSS receiver obtaining correction data from the TopNet (Czech provider of correction data). With the same equipment, the position of the GNSS base station (Figure 7) was determined. The base station, also indicated within Figure 6, provides the correction data for the GNSS receiver aboard the UAS, and its accurate position is crucial as regards the quality of the snow-field parameters estimation.

### 2.5. Mission Planning and Aerial Data Acquisition

Mission planning comprises standard tasks known from UAS-based photogrammetry. First, a mapping region must be selected; in our experiment, this was a rectangular area around the isolated snow field. Both horizontal coordinates of the region are enlarged by approximately 50% because the exact size and location of the snow field during the melting period is difficult to estimate. Subsequently, we design the trajectory (the waypoints for the automatic flight) within the region, satisfying the following photogrammetric requirements: the desired forward and side image overlaps; ground resolution; and altitude restrictions. The trajectory planning thus also depends on the applied photographic equipment, its intrinsic parameters in particular; these include, for example, the principal distance and sensor size. We use the common parallel strips flight pattern known from both manned and unmanned aerial photogrammetry [39,40], as shown in Figure 6. The parameters of the flight trajectory and image data acquisition for the presented case study are summarized in Table 2.

The mission planning process is, in our case, performed in the field, immediately after assessing the actual situation. For this purpose, a laptop with the MikroKopter-Tool software for automatic waypoint-based mission planning is used. Since the location is a steep slope (the height difference between the highest and lowest spots is approximately 100 m), a constant flight altitude AMSL would cause high variation in altitude AGL and thus also ground resolution. For this reason, we adjusted the AMSL altitude of the individual survey lines to minimize variation in ground resolution.

Except for the take-off and landing, the UAS operates in the automatic mode based on the aforementioned settings, uploaded to the device’s memory just before the flight. With the employed photogrammetric parameters, the mapping of the area takes approximately 10 min. The image and GNSS/INS data, which are hardware-synchronized, are recorded throughout the flight on the SD card and SSD storage, respectively.

### 2.6. Photogrammetric Processing

The method proposed in this paper uses photogrammetry to reconstruct the given 3D scene, namely snow-covered terrain. For this purpose, we used Agisoft Photoscan Professional (version 1.4.2), a complex software package to execute all photogrammetric processing stages, from the image and georeferencing-related data to the orthophoto, DEM, and other products. The workflow starts with the align phase, where the exterior and interior camera orientation [41] is estimated based on the feature points detected in the overlapping images. Furthermore, the locations of the feature points are determined via the structure-from-motion procedure, resulting in a sparse point cloud [42]. Within the following step, a dense point cloud can be generated, using multi-view stereo (MSV) reconstruction. As the operation is performed at the level of pixels, even small details are reconstructed to yield a point cloud containing millions of points.

The camera poses determined in the previous step being relative, Photoscan offers two methods of georeferencing, namely transformation into geographic coordinates (WGS-84 in our case): one employing the image position data measured by the onboard sensors (Section 2.3), and the other performed via the GCPs positions obtained during a ground measurement (Section 2.4). Both procedures rely on similarity transformation comprising translation, rotation, and scaling [39,43]. The techniques are also known as direct (DG) and indirect georeferencing (IG), respectively. Although we used a multi-sensor system to carry out the GCP-free photogrammetry, both georeferencing options were tested. For this reason, the georeferencing phase of the workflow was executed twice to evaluate the two methods separately (employing the same image dataset and identical photogrammetric processing settings). In practice, most of today’s photogrammetry missions use GCPs to reach a centimeter-level spatial accuracy even with consumer-grade equipment onboard a UAS; this scenario is presented in several sources, such as [22,44,45]. DG, commonly supported by computer vision (CV) algorithms, typically leads to a slightly lower accuracy; this approach, however, brings certain benefits, as outlined within the above chapters and elsewhere [22,23,24].

Based on the georeferenced dense point cloud, a DEM was generated; the model can be considered a digital surface model (DSM). This product is essential for the formation of the orthophoto. The quality of these outputs was determined by using test points, or ground targets with accurately known spatial positions (Section 2.4). We used the targets in two manners, namely as the TPs in DG and the GCPs in IG. The majority of the referenced papers employed Photoscan, whose workflow and algorithms are described in more detail within [46].

Photogrammetry-based 3D scene reconstruction fully depends on the features visible in the images, resulting in certain restrictions as regards the presented study. Snow-covered terrain typically lacks a texture because a snow surface normally contains white color that does not vary substantially. Such a property then causes the reconstruction process to fail; however, relevant studies show that snow often contains a texture sufficient for photogrammetric processing [18,20,21]. Finally, low visibility caused by weather effects, including fog or clouds, will also produce a negative impact on the quality of the results.

### 2.7. Snow-Field Parameter Estimation

For many UAS-based mapping applications, the final output consists of photogrammetry products, such as a georeferenced orthophoto and DEM. However, in our case, these products form the input data for the final processing phase, namely the calculation of the snow-field parameters (see the process flow diagram in Figure 4). As described in the Introduction, the three main parameters related to snow mapping are SCA; SD, or snow volume; and SWE. Our method considers merely the first two of these as the last one typically requires in situ measurements.

**Figure 8:**
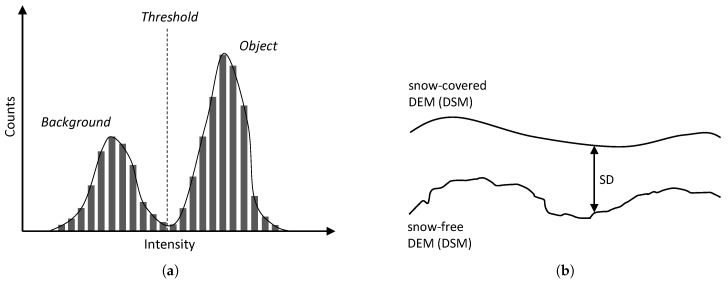
Segmentation by thresholding: a grayscale image histogram and the threshold separating an object and the background (**a**); the snow depth determination principle (**b**).

The method uses optical-based SCA estimation employing the orthophoto obtained within the previous step. This approach relies on visual separability of the snow cover from the rest of the snow-free terrain, typically covered with vegetation, rocks, water, or artificial objects. The desired effect is achieved through segmentation, an elementary CV task that consists of partitioning an image into multiple homogeneous and meaningful regions [47]. A common segmentation technique is thresholding [48], which in its basic form, separates an object and the background in a grayscale image. The key assumption is that the object class includes levels of gray different from those exhibited by the background class, enabling the user to find the thresholding level (Figure 8a).

The snow field (representing the object, with a color approaching white) in our study area is clearly separable from the snow-free terrain (or the background, covered with vegetation) via thresholding since the two classes are characterized by different levels in both red-green-blue (RGB) channels and the grayscale interpretation. Within our research, the threshold level was established primarily manually to facilitate precise SCA determination; we nevertheless also employed the well-known Otsu method [49] (implemented within the graythresh function in MATLAB) to test the automatic threshold level determination. Once the snow has been separated from the background, the snow field, Mapa Republiky, must be separated from the snow cover in its vicinity. This step was performed using the bwlabel function, which labels connected components in the binary image. The largest component then corresponds to the snow field, and its area is determinable thanks to the known pixel size of the orthophoto.

The aforementioned semi-automatic and automatic SCA estimation methods were also compared with the manual technique exploiting an area measurement tool contained in geographic information systems (GISs); this solution allows the operator to measure any area manually. All the approaches are discussed in the final sections of this article.

**Figure 9:**
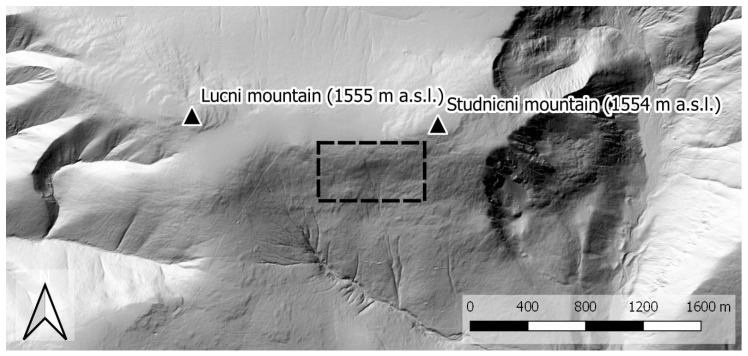
The fifth generation, shaded DTM of the Krkonose (obtained from CUZK). The study area is highlighted by the dashed line.

Snow depth, expressible with a positive real number, describes the height of the snow cover above the terrain at a certain location; a snow depth map is then the 2D SD representation of the given area georeferenced into geographic coordinates. SD, unlike the above-described SCA, cannot be estimated using an orthophoto only. Our method interprets SD as the height difference between a snow-covered and a snow-free DEM, as illustrated in Figure 8b. The former model was obtained during a melting season via photogrammetry (Section 2.6); the latter, snowless elevation model was acquired from CUZK (the Administration of Land Surveying and Cadastre of the Czech Republic [50]). The Administration provides an airborne laser scanning (ALS)-based digital terrain model (DTM) covering the entire Czech Republic; its height accuracies are 0.15 m and 0.25 m for uncovered and wooded terrain, respectively, and the average density corresponds to 5 points m${}^{-2}$ in the very least. This model was resampled to obtain a DTM with the resolution of 0.05 m px${}^{-1}$ (the shaded DTM of the study area is illustrated in Figure 9). Unlike the DSM produced by photogrammetry, the DTM does not involve artificial objects and vegetation. However, this property should not lead to major difficulties, because our study area is mostly covered with grass, and no objects are present. The calculation was performed in QGIS (version 3.0.2) GIS to allow calculations with raster layers and to support various coordinate reference systems. The input raster layers, or DEMs, were obtained using different methods; thus, their pixel resolutions do not coincide. To deal with this issue, the nearest neighbor resampling method was applied.

The drawback of the SD calculation technique is that the height difference between the DEMs does not have to be caused by the snow only. In mountainous regions, a rapid surface change can occur due to, for example, vegetation growth or a rock slide. To minimize the impact of such effects, we consider only areas where the presence of the snow was detected using an orthophoto, as described within the SCA determination earlier in this section. The masked (snow-free) area is thus excluded from the SD calculation.

The snow-cover volume is another meaningful indicator. As with the SCA, it informs us about the quantity of snow via a single number. Once the SD map has been obtained, the volume can be computed as the sum of the individual heights multiplied by the map resolution (pixel size). From this point, there remains only a small step to determining the SWE, namely the amount of water contained in the snow field. Since the snow density oscillates between 50 kg m${}^{-3}$ (new snow) and 900 kg m${}^{-3}$ (ice, [51]), the parameter must necessarily be obtained. Such a task, however, is not feasible using the sensors onboard our UAS; to obtain relevant data, we would therefore have to rely on in situ observation.

## 3. Results

### 3.1. Photogrammetry

**Figure 10:**
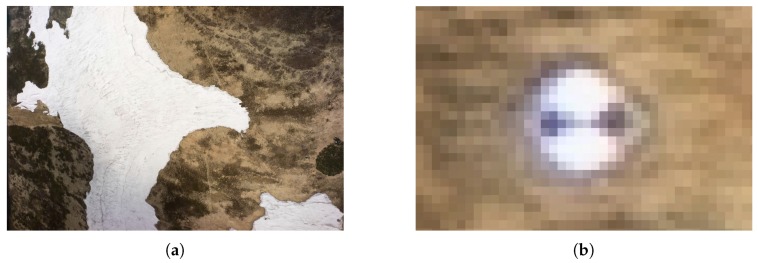
A photo captured from the height of 100 m AGL (**a**); a zoomed ground target (**b**).

The aerial data acquisition lasted 14 min; during this time, 403 images were collected. In addition to the automatic waypoint-based flight, the operational period included the take-off and landing, namely manually controlled phases. The data acquired within these latter steps are not relevant; thus, 222 images only were used for the processing. This image set was preprocessed using Adobe Photoshop Lightroom (version 6.0) to compensate lens vignetting and to convert the RAW image files into JPG at minimum compression. One of the images is displayed within Figure 10a.

**Figure 11:**
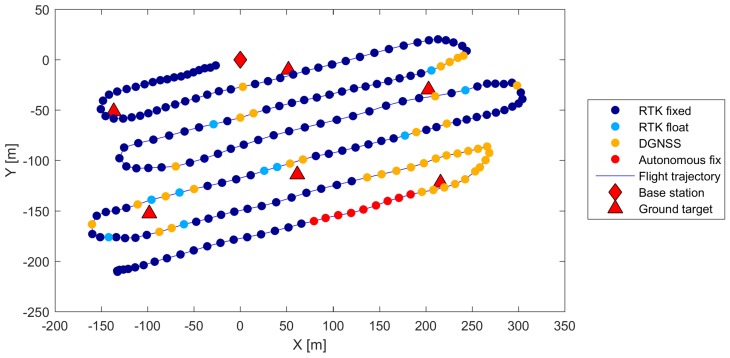
The camera locations and the GNSS quality indicator estimated by the onboard GNSS/INS.

The GNSS/INS logging was triggered with the camera shutter, and the number of records contained in the log file equaled that of images captured. The data were converted into an appropriate format and subsequently analyzed. Figure 11 shows the measured positions of the cameras in the local system as well as the solution quality indicator estimated by the onboard GNSS. The RTK fixed solution was available for 73% of the time; the RTK float for 5%; the differential GNSS (DGNSS) for 18%; and the autonomous fix for 4%. The RTK fixed solution outages were caused by interruption of the data link that facilitates the transmission corrections. The age of the corrections has a direct impact on the quality of the GNSS solution; the fixed solution was thus lost for several moments, especially in the southeastern part of the flight trajectory, as is evident from Figure 11. This fact will likely negatively affect the accuracy of the DG and, therefore, also that of the photogrammetric products. The aforementioned figure further comprises the actual locations of the GCPs employed for the IG; one of the targets (as captured in an aerial image) is displayed within Figure 10b.

The datasets from the onboard sensors, namely the images and the GNSS/INS derived data were imported into the Photoscan, together with the locations of the ground targets. The photogrammetric processing was performed once with DG and once with IG, using the same settings. These techniques, except for georeferencing itself, require identical processing phases. In the case of DG, the camera locations (and estimated errors) were simply imported from a text file; IG, conversely, involves manual placement of markers on the targets visible in the images. Despite this, the processing times do not differ much, as indicated in Table 3. However, if we also consider the data collection phase, IG is more time-intensive due to the deployment of ground targets (GCPs).

**Figure 12:**
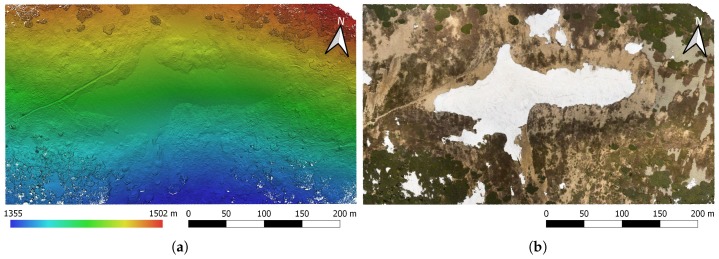
The directly georeferenced, shaded DEM (**a**) and the relevant orthophoto (**b**), both representing the output of the photogrammetric processing phase.

The parameters of the photogrammetric products are also comparable to a certain extent: Both dense point clouds contain approximately 190 points m${}^{-2}$, the resolutions of the DEMs and the orthophotos are 10.9 cm px${}^{-1}$ and 2.7 cm px${}^{-1}$, respectively (Figure 12). As expected, the difference shows itself in the georeferencing accuracy. According to the 6 TPs, DG reached the spatial RMS error of 11.4 cm; the RMSE of IG is slightly better, equaling 7.6 cm (Table 4). It should be noted that although using such a low number of TPs to express accuracy is not sufficiently credible in terms of statistics, we can still benefit from the actual information of whether a problem occurred during the data collection and processing. A detailed analysis of DG accuracy was proposed within our previous study [36]. Furthermore, IG accuracy stated using GCPs (with the targets employed for georeferencing) is also of mainly informative character.

The analysis indicates that in our study, the georeferencing techniques do not differ significantly as regards the time intensity and the final accuracy (IG needed 34% more time, Table 3; DG was slightly less accurate, Table 4). The main benefit of DG rests in the elimination of human involvement, especially during the data collection procedure, which is potentially hazardous to human health. Moreover, such a scenario enables automation of all major data collection and processing phases.

### 3.2. Snow-Field Parameters

**Figure 13:**
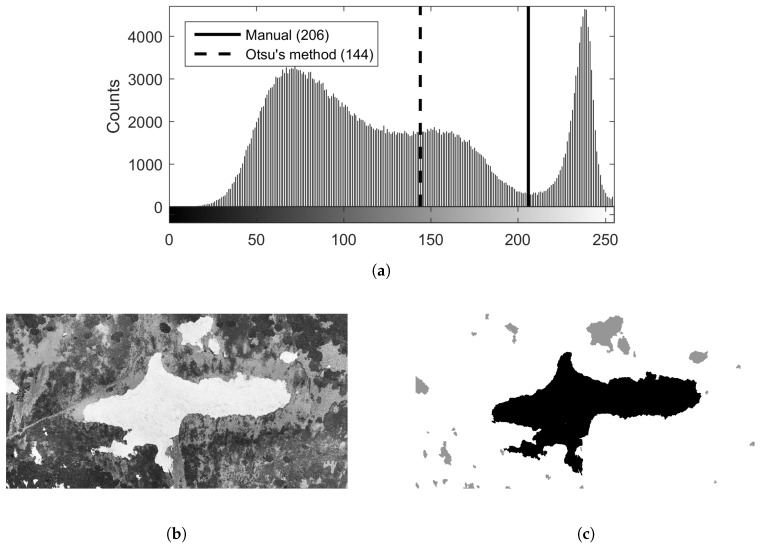
The histogram (**a**) of the gray-scaled orthophoto (**b**), with the snow-covered area extracted (the snow field is highlighted in black) (**c**).

The calculation of the snow-field parameters started with the orthophoto-based SCA estimation, as discussed in Section 2.7. First, the orthophoto was converted into an 8-bit grayscale image (Figure 13b) for segmentation purposes. We determined two threshold levels in the respective histogram: one level, 206, was established manually, while the other, 144, was computed using the Otsu method (Figure 13a). The Otsu technique was found to produce unsatisfactory results in this application as it does not separate the object from the background precisely; the threshold should lie in the local minimum between the high valued counts (whites) and the darker background. To automate this procedure, a reliable segmentation algorithm would have to be designed; such a step, however, would require an adequate test dataset. For this reason, we applied the manual threshold level. In the segmented image, connected components were found; the component comprising the largest number of pixels corresponded to the snow field (Figure 13c). Based on the known pixel size, the SCA of 15,802 m${}^{2}$ was estimated. This result was then manually verified using the QGIS area measurement tool, yielding 15,493 m${}^{2}$, a value 2% lower than the automatically delivered one.

**Figure 14:**
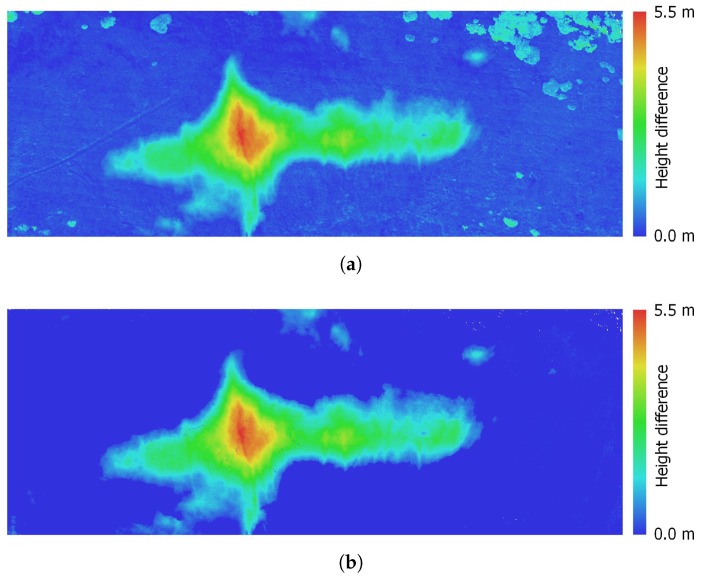
The height difference between the snow-covered, photogrammetry-based DSM and the snow-free, ALS-based DTM (**a**). This result involves differences caused by not only the snow itself but also other aspects, including, for example, vegetation (upper right corner). After the SCA mask has been applied, the difference map contains the SD only (**b**).

The SD map was derived from the elevation models. Since both the snow-free ALS-based DTM and the snow-covered photogrammetry-based DSM are georeferenced in the same coordinate system, they were used for direct calculation. At the initial stage, we employed the QGIS raster calculator to compute the height difference between the DSM and the DTM. Since the former, unlike the latter, generally also includes vegetation and artificial objects, the difference map does not contain the snow only. This effect is represented in Figure 14a, where, especially in the upper right corner, the vegetation (scrub mountain pine) causes considerable height differences. To exclude such objects from the SD calculation, we applied the SCA mask computed within the previous step. After that, the difference map contained the snow cover only, as displayed in Figure 14b. The maximum height of 5.45 m corresponds to the maximum depth of the Mapa Republiky snow field, whose volume, computed as the sum of the heights in the individual pixels, equals 26,367 m${}^{3}$.

As is evident, the study area contains various types of vegetation. Since we used the DTM as the snow-free reference, it cannot be excluded that some of the vegetation is under the snow cover. Such a scenario would negatively affect the accuracy of the SD determination, meaning that the estimated SD would be higher than it really is. To deal with this issue, a DSM created via, for example, UAS-based photogrammetry, should be employed as the snow-free reference model.

**Figure 15:**
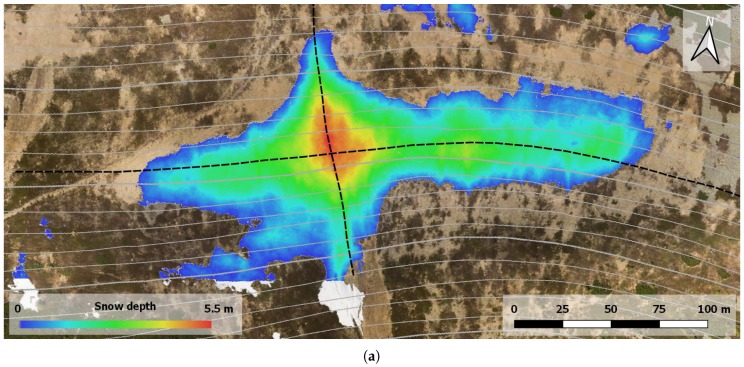

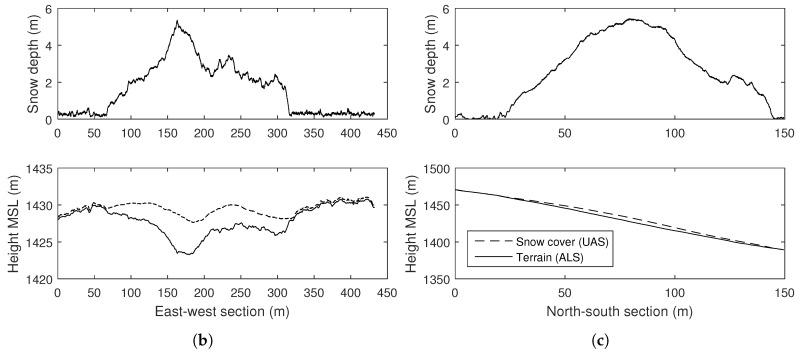
An interpretation of the mapping results (**a**). The UAS photogrammetry-based orthophoto is supplemented with a layer illustrating the snow depth; both items were created with the multi-sensor system for direct georeferencing. The map includes contour (solid gray) and section (dashed black) lines to facilitate the cross-section analysis. The cross-sections of the SD map, the UAS-based surface model, and the ALS-based terrain model, all passing through the SD maximum in the east-west direction, are displayed in (**b**); those that run in the north-south direction are then indicated in (**c**).

The high-resolution models introduced within our research allow us to analyze cross-sections in any direction, leading to better understanding of how the snow is accumulated in relation to the terrain shape. An example of two cross-sections through the SD maximum in the east-west and north-south directions is provided in Figure 15. The east-west direction, for instance, illustrates that the maximum is located exactly in the terrain depression present within the area of interest.

## 4. Discussion

Within this study, we introduced an innovative, state-of-the-art technique for snow-field monitoring and tested its performance at Mapa Republiky, a location of major scientific interest within the discussed research problem. The method exploits various advanced technologies, such as micro UASs to perform automatic aerial data collection; an embedded RTK GNSS receiver allowing centimeter-level positioning; and photogrammetric software providing powerful tools to create digital orthophotos and elevation models. Our solution thus effectively eliminates most drawbacks inherent in previous approaches to the estimation of SD, volume, and covered area.

In the context of the mapping, we can specify several categories to evaluate the quality of each method, and these are as follows: spatial density (resolution) of the collected data; SD estimation accuracy; data acquisition time; safety risks; and automation potential. We comprehensively evaluated each of the methods employed previously on Mapa Republiky as well as the procedure proposed herein; the results are summarized in Table 5. As regards the earliest technique, which relied on wire probes, the main drawbacks consisted of the impossibility to penetrate thick snow layers (above 3–4 m); very long measurement time; and low spatial density. The SD estimation accuracy is sufficient (less than 10 cm, as established upon comparison with the GNSS-based method [26]) if the probe reaches the ground. As with all methods requiring manual data collection, wire probing is a low safety procedure due to the avalanche risk. The approach shares most of its parameters with the GNSS-based ground procedure, introduced within [26,31]; significantly, the latter method is nevertheless capable of measuring thick snow layers without accuracy reduction. Since the data collection is realized through continuous walking or skiing, higher space density data can be obtained in less time. Furthermore, a rope-driven sledge can be used instead of the walking and skiing to decrease the safety risk during the avalanche season.

The UAS-based aerial data acquisition markedly reduced the data collection time without decreasing the accuracy and, simultaneously, ensured superior density (at the centimeter level). In the remaining categories, the quality varies with the applied georeferencing technique. As mentioned in Section 2.6, the IG in UAS-based photogrammetry outperforms the direct technique in terms of the object accuracy. Both approaches, however, allow us to reach a centimeter-level object accuracy [22,32,52]. This fact was confirmed during our previous research [36] and within this study as well. We achieved the spatial RMS error of 11.4 cm in the DG, securing sufficient performance for the relevant application, given that the height accuracy of the applied snow-free model was 15 cm RMS. Yet, the SD estimation accuracy was not assessed in this study; this topic is discussed, for example, within articles [7,8]. IG, compared to DG, does not require very accurate and expensive positioning equipment onboard the UAS; despite this, a survey-grade GNSS receiver is still needed to measure the positions of the GCPs, an operation that consumes substantial extra time (Table 3 and [7]). In the remaining respects, DG nevertheless brings a major advantage in that the field work comprises only the automatic UAS flight, enabling the operator to control the entire mission from a safe distance. Such a scenario then considerably reduces the health risks and time intensity. Additionally, DG enables us to significantly increase the portion of automatic processing. In terms of the aforementioned aspects, the method that uses UAS-based photogrammetry and DG can be considered the most appropriate solution for snow mapping at present.

In the context of Mapa Republiky, our approach brings undoubtable benefits, the most prominent one being that the snow monitoring can begin already in early spring, namely at a time when the avalanche risks are usually too high to employ other techniques, terrestrial-based procedures in particular. This advantage enables us to collect complex data over a longer time period, leading to a better insight into the snow melting process and its impact on the local microclimate and hydrological situation. Moreover, the lower time intensity and higher automation potential may facilitate more frequent observations in the future. The method’s usability, however, is not limited to the discussed site only: Many other research-relevant, interesting locations in the Krkonose cannot be easily monitored by UASs today, mainly due to the avalanche risk, inaccessibility, or dense vegetation disallowing GCP deployment.

The only partially unsuccessful portion of the experiment was the correction transmission during the aerial data acquisition, where the datalink outages probably slightly affected the object accuracy. As regards the data processing, the segmentation phase will have to be refined to eliminate operator interventions; we nevertheless believe that the entire data processing cycle can be executed automatically in the future.

It should be noted that the proposed approach exhibits certain limitations. The operation of the UASs, the small ones in particular, depends on the weather conditions: Flying in rain or snowfall is typically prohibited, and the wind speed cannot exceed the pre-defined level. The discussed conditions may embody a markedly limiting factor, especially in mountainous regions and during the winter season, namely parameters specific to our research. Moreover, poor visibility (due to fog, for example) can affect the quality of the photogrammetric processing or even make this phase impossible.

Further development of the technology will most likely increase the already high effectivity of mapping missions. Thus, our solution relying on a custom-built multi-sensor system and a UAS, with the total weight of the components being approximately 8 kg, may be possibly replaced with a more compact design. RTK GNSS-equipped UASs whose weight ranges below 2 kg have been recently available on the market [53]; using such equipment, the field work could be easily carried out by one person. The quality of this solution, however, is yet to be assessed.

## 5. Conclusions

This article proposed a UAS-based snow-field mapping technique and evaluated its performance at Mapa Republiky, a mountainous location of substantial scientific interest. The main benefit of the method rests in using ground control point-free UAS photogrammetry enabled by a custom-built multi-sensor system; this approach allowed us to perform an entire mapping mission from a safe distance, eliminating a major portion of the safety risks associated with avalanches. The parameters observed within the experiment, namely the georeferencing accuracy (approximately one decimeter RMSE) and spatial resolution of the photogrammetric products (centimeter-level), fully satisfied the requirements of the discussed application. Regrettably, the thick snow cover at the study site did not enable us to evaluate the SD mapping results achievable with a direct method, such as wire probing. In terms of the time consumption, our approach proved to be more efficient than the previously employed techniques, facilitating automatic execution of most processing steps. Despite this, there remains space for improvements, especially in view of the fact that some processing procedures currently depend on user intervention. Exploiting the outcomes presented herein, those related to safety in particular, the technique generally allows the user to collect data at Mapa Republiky very early in spring; such an operation was not feasible previously, meaning that the associated microclimate research at the given location had to face substantial objective limitations. This impediment can now be effectively overcome by using the novel approach.

## Figures and Tables

**Table 1:** The parameters of the custom-built multi-sensor system for UASs. The position and attitude accuracy according to the INS manufacturer’s specifications (the RTK mode, airborne applications).

Parameter	Value
Position accuracy	hor.: 20 mm, ver.: 40 mm
Attitude accuracy	roll/pitch: 0.1${}^{\circ }$, heading: 0.4${}^{\circ }$
Camera sensor resolution	6000 × 4000 px
Camera sensor size	36 × 24 mm
Camera principal distance	21 mm
Camera aperture	f/4.5
Operational time	120 mins
Max. distance from base	1000 m
Dimensions	1.5 × 0.2 × 0.2 m
Weight	2.6 kg

**Table 2:** The parameters of the flight trajectory and image acquisition; the values that depend on the flight altitude (AGL) are stated for the average altitude.

Parameter	Value
Distance between strips	30 m
Strip length	400 m
Number of strips	6
Base (distance between consecutive images)	10 m
Flying altitude AGL	90–130 m
Flying speed	5 m s${}^{-1}$
Flying time	10 min.
Time between images	2 s
Photo scale	1:5200
Forward overlap	92%
Side overlap	84%
Image footprint	190 × 125 m
Ground resolution	3.1 cm px${}^{-1}$
Shutter speed	1000${}^{-1}$ s
Aperture	5.6
ISO	Auto (100–400)

**Table 3:** The data collection and processing times of the applied techniques; the photogrammetric processing was executed on a personal computer with an Intel Core i7-6700 CPU, 32 GB RAM, and an NVIDIA GeForce GTX 1051 Ti GPU.

Processing Phase	Automatic/Manual	DG [h:mm:ss]	IG [h:mm:ss]
Aerial data acquisition	A	0:13:36	0:13:36
Target deployment	M	—	~0:30:00
Data collection in total	—	0:13:36	~0:44:00
Photo alignment	A	0:18:33	0:21:00
Marker placement	M	—	0:09:55
Camera calibration	A	0:00:06	0:00:06
Dense point cloud generation	A	1:23:59	1:25:33
DEM generation	A	0:00:36	0:00:26
Orthophoto generation	A	0:09:36	0:08:04
Processing in total	—	1:52:50	2:05:04
Entire process	—	2:06:26	2:49:04

**Table 4:** The accuracy of direct and indirect georeferencing, determined by using 6 ground targets. In the IG procedure, the accuracy was assessed via the GCPs.

	DG [cm]			IG [cm]		
Target	XY	Z	XYZ	XY	Z	XYZ
1	1.3	−1.3	1.8	4.1	−7.7	8.8
2	2.7	2.8	3.9	1.8	6.6	6.9
3	2.2	−0.3	2.2	3.2	3.3	4.6
4	7.5	−6.4	9.9	5.2	−6.6	8.4
5	21.8	10.7	24.3	10.0	1.5	10.1
6	3.5	7.7	8.5	5.6	2.2	5.6
Mean	6.5	2.2	8.4	4.9	−0.1	7.4
RMSE	9.7	6.1	11.4	5.5	5.3	7.6

**Table 5:** A comparison of the snow-field mapping methods.

Type	Method	Resolution (Meter-Order)	Accuracy (Meter-Order)	Time Consumption	Safety	Automation Potential
Ground	wire probe survey	10${}^{0}$–10${}^{1}$	10${}^{-1}$–10${}^{0}$	hrs	avalanche risk	no
Ground	GNSS survey	10${}^{0}$–10${}^{1}$	10${}^{-2}$	hrs	avalanche risk	no
Ground + aerial	UAS photo. (IG)	10${}^{-2}$	10${}^{-2}$	mins–hrs	avalanche risk	partial
Aerial	UAS photo. (DG)	10${}^{-2}$	10${}^{-2}$–10${}^{-1}$	mins	no	high
